# The Recombinant Maize Ribosome-Inactivating Protein Transiently Reduces Viral Load in SHIV89.6 Infected Chinese Rhesus Macaques

**DOI:** 10.3390/toxins7010156

**Published:** 2015-01-19

**Authors:** Rui-Rui Wang, Ka-Yee Au, Hong-Yi Zheng, Liang-Min Gao, Xuan Zhang, Rong-Hua Luo, Sue Ka-Yee Law, Amanda Nga-Sze Mak, Kam-Bo Wong, Ming-Xu Zhang, Wei Pang, Gao-Hong Zhang, Pang-Chui Shaw, Yong-Tang Zheng

**Affiliations:** 1 Key Laboratory of Animal Models and Human Disease Mechanisms of Chinese Academy of Sciences & Yunnan province, Kunming Institute of Zoology, Chinese Academy of Sciences, Kunming 650223, Yunnan, China; E-Mails: wangrr1980@163.com (R.-R.W.); hongyzheng@163.com (H.-Y.Z.); snoopykm@126.com (X.Z.); luorh@mail.kiz.ac.cn (R.-H.L.); mingxu2008-2009@163.com (M.-X.Z.); pangw@mail.kiz.ac.cn (W.P.); zhanggh@mail.kiz.ac.cn (G.-H.Z.); 2 Center for Protein Science and Crystallography, School of Life Sciences, the Chinese University of Hong Kong, Shatin N.T. 999077, Hong Kong, China; E-Mails: aaky73@yahoo.com.hk (K.-Y.A.); sue.suelaw@gmail.com (S.K.-Y.L.); amandamak@gmail.com (A.N.-S.M.); kbwong@cuhk.edu.hk (K.-B.W.); 3 Division of HIV/AIDS and STI Control, Yuxi Centers for Disease Control and Prevention, Yuxi 653100, Yunnan, China; E-Mail: glmcharge2012@gmail.com

**Keywords:** maize RIP, anti-HIV, animal model, viral load

## Abstract

Ribosome inactivating proteins (RIPs) inhibit protein synthesis by depurinating the large ribosomal RNA and some are found to possess anti-human immunodeficiency virus (HIV) activity. Maize ribosome inactivating protein (RIP) has an internal inactivation loop which is proteolytically removed for full catalytic activity. Here, we showed that the recombinant active maize RIP protected chimeric simian-human immunodeficiency virus (SHIV) 89.6-infected macaque peripheral blood mononuclear cells from lysis *ex vivo* and transiently reduced plasma viral load in SHIV89.6-infected rhesus macaque model. No evidence of immune dysregulation and other obvious side-effects was found in the treated macaques. Our work demonstrates the potential development of maize RIP as an anti-HIV agent without impeding systemic immune functions.

## 1. Introduction

Ribosome inactivating proteins (RIPs) are RNA *N*-glycosidases. They cleave the *N*-glycosidic bond of adenine-4324 located at the GAGA hairpin of the sarcin/ricin loop of 28S rRNA and stop protein synthesis.

RIPs are therefore highly cytotoxic and they have been developed into immunotoxins, anti-viral and anti-tumor agents [1,2,3]. RIPs are classified into three types according to the number of subunits and the existence of an internal inactivation loop. Type 1 RIPs, such as trichosanthin (TCS) and pokeweed antiviral protein (PAP), exist as a single polypeptide. TCS has been used in Chinese folk medicine for centuries to terminate gestation at early or mid-term stages and to treat hydatidiform moles [4] which may be explained by the high sensitivity of syncytiotrophoblasts towards the cytotoxic RIPs and undergo fragmentation [5]. It is well-known that type 1 RIPs possess anti-HIV properties. Three PAP isoforms damaged HIV-1 genome by concentration-dependent depurination of the viral RNA [6]. Saporin and luffin inhibited HIV-1 integrase and prevented insertion of viral DNA into the host’s chromosome [7]. TCS was found to bind and depurinate HIV-1 long terminal repeats (LTR) which are sites important for provirus integration [8]. *Gelonium* anti-viral protein 31 (GAP31) [9,10] and *Momordica* anti-HIV protein 30 (MAP30) [11,12] also inactivated viral DNA by changing the topological property of HIV-1 LTR and in turn inhibited viral DNA integration. Clinical tests of TCS and PAP were launched to evaluate their efficacy for treating HIV patients. The patients with AIDS or AIDS-related complex were recruited and administrated weekly with TCS by intravenous infusion at 36 or 50 µg/kg for four consecutive weeks. Increases in CD4^+^ and CD8^+^ T cells were observed with moderate side effects of myalgia and flu-like syndrome and the rise of CD4^+^ T cells was shown to sustain for at least 28 days after the last infusion [13]. Immunotoxin of PAP conjugated with antibodies for CD7 (TXU-PAP) was assessed clinically and shown to be well tolerated by HIV-1-infected adult patients after a single intravenous infusion at 5 µg/kg. TXU-PAP was capable of lowering the viral load in all patients evaluated without any adverse reactions and plasma p24 antigen reduction of over 50% was detected in one individual [14]. Type 2 RIPs, such ricin, consist of an enzymatically active A chain similar to type 1 RIPs linked to a lectin-like B chain by a disulphide bond. With the B chain that directs ricin A chain to the cytoplasm, where ribosomes situate, for rRNA depurination to cause consequent cell death [1,15], ricin is one of the most toxic substances in the world, with the lethal dose of 350–700 µg for a 70 kg human upon inhalation or injection [16].

Maize RIP is classified as type 3 RIP which is first synthesized as an inactive precursor containing an internal inactivation loop and requires proteolytic removal of the fragment to produce a heterodimer with full *N*-glycosidase activity. Our group has solved the structure of maize RIP to 0.25 nm resolution [17]. The inactivation loop was revealed to protrude out on the surface of the protein and sterically block the interaction with ribosome, making its removal necessary to resume rRNA depurinating action. We also reported the active maize RIP suppressed viral replication in HIV-1 acutely infected C8166 cells with EC_50_ values of 0.21–0.62 µM, whereas the inactive precursor had limited inhibition [18].

With positive results showing the *in vitro* HIV-inhibitory activity of maize RIP in T lymphocyte cell lines, we want to know if this type 3 RIP also possesses antiviral activity *in vivo* and is safe for clinical tests. In this study, the anti-HIV effect of maize RIP is assessed in simian immunodeficiency virus (SIV) and chimeric simian/human immunodeficiency viruses (SHIV)-infected macaque peripheral blood mononuclear cells (PBMC) and *in vivo* using SHIV 89.6-infected Chinese rhesus macaque model. Parameters including complete blood count, liver injury and weight are also closely monitored to evaluate the drug safety of maize RIP for animal studies.

## 2. Results

### 2.1. Antiviral Effects of Maize RIP on Infected Macaque PBMC

**Figure 1:**
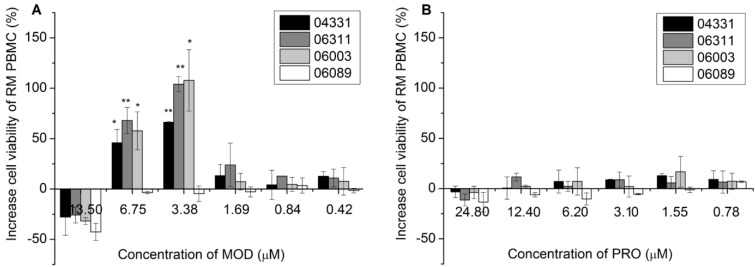
Protection for simian-human immunodeficiency virus (SHIV)-induced lysis on SHIV89.6-infected macaque PBMC upon treatment of (**A**) His-TAT-MOD; and (**B**) His-TAT-Pro. The experiment was repeated three times and mean ± SD was calculated for graphic presentation. Paired *T*-test was used for statistical analysis (* *p* < 0.05, ** *p* < 0.01).

The antiviral efficacies of maize RIP were assessed *ex vivo* on macaque PBMC by protection assay. His-TAT-MOD was shown to increase the viability of PBMC isolated from SHIV89.6-infected macaques (#04331, #06003 and #06311) by 50% and 100% at the non-cytotoxic doses of 3.38 and 6.75 µM, respectively, whereas a similar observation was not detected on uninfected cells (#06089) (Figure 1A). The precursor His-TAT-Pro showed no protective effect on healthy or infected PBMC (Figure 1B). The exclusive observation that the active form of maize RIP differentially enhanced the cell survival of infected PBMC, but not healthy PBMC, hints that the protective effect is attributed to the antiviral property of His-TAT-MOD.

The antiviral efficacies of maize RIP were also assessed *in vitro* on macaque PBMC. His-TAT-MOD reduced the viral antigen production in SHIV89.6- and SIVmac239- infected PBMC with 50% effective concentration (EC_50_) at 5.53 and 11.23 µM, respectively whereas the precursor had limited antiviral effect (Table 1).

**Table 1:** Cytotoxicity and antiviral activities of maize ribosome inactivating protein (RIP) variants tested on rhesus macaque peripheral blood mononuclear cells (PBMC).

RIP variants	Cytotoxicity CC_50_ (μM)	p27 Antigen reduction EC_50_ (μM)	
	Uninfected	SHIV89.6	SIVmac239
His-TAT-Pro	>15	>24.85	>24.85
His-TAT-MOD	8.98 ± 0.36	5.53	11.23

### 2.2. Antiviral Activities of Maize RIP in Rhesus Macaques

**Figure 2:**
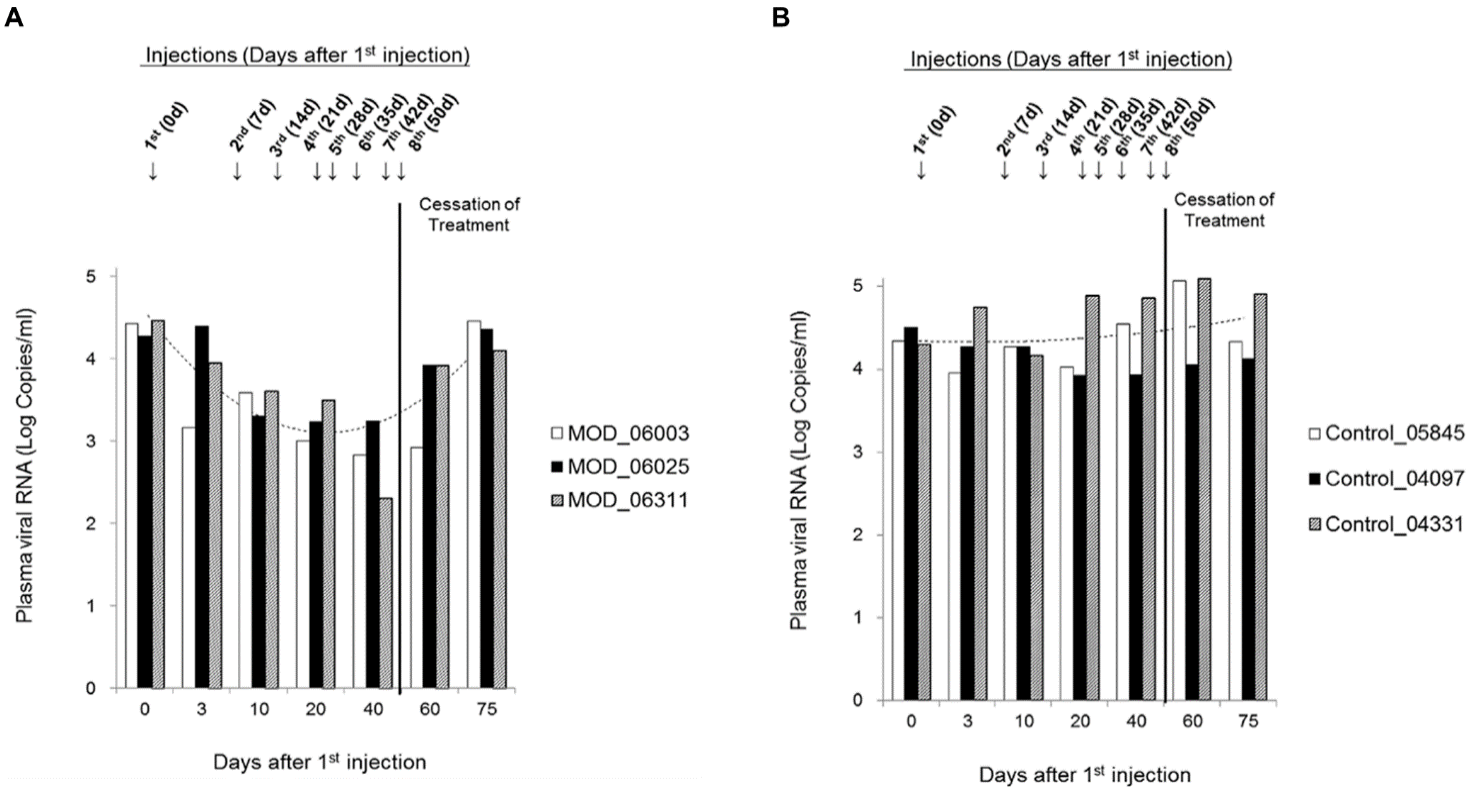
Plasma SIV viral load in rhesus macaques treated with (**A**) His-TAT-MOD; and (**B**) normal saline as negative control. Each group consisted of three macaques and “---” represented trend-line plotted using the average value from three individuals.

Chinese rhesus macaques were employed as non-human primate model for assessing the antiviral efficiency of maize RIP *in vivo*. SHIV89.6-infected macaques were administered with either His-TAT-MOD or normal saline as negative control and had the plasma viral load determined. During treatment, the viral load of MOD-treated group decreased gradually and had an overall drop of 30% by the end of treatment period (40 d; Figure 2A) whereas that of control group remained stable (40 d; Figure 2B). After cessation of treatment, plasma viral load of MOD-treated group rose again and on 75 d after first injection (25 d post-treatment), the level was comparable to the initial value (75 d; Figure 2A). Statistical analysis confirmed the association between His-TAT-MOD treatment and the decrease in plasma SIV load (*p* < 0.001).

Table 2Hematological parameters in macaques administrated with (**A**) His-TAT-MOD (*n* = 3); and (**B**) normal saline (*n* = 3). Abbreviations: ALT, alanine transaminase; AST, aspartate transaminase; WBC, white blood cells; Lym, lymphocytes; Mon, monocytes; Gran, granulocytes; RBC, red blood cells; HGB, haemoglobin; HCT, haematocrit; MCV, mean corpuscular volume; MCH, mean corpuscular haemoglobin; PLT, platelets; MPV, mean platelet volume.

**(A):** 

Markers	Days after first injection of His-TAT-MOD							
	0	3	10	20	40	60	75	*p* value
**ALT (U/L)**	41.67 ± 31.07	40.33 ± 32.08	41.33 ± 36.02	28.33 ± 19.40	24.00 ± 16.70	21.67 ± 16.01	22.00 ± 17.09	0.022
**AST (U/L)**	24.67 ± 15.63	34.67 ± 21.39	28.00 ± 23.90	29.00 ± 20.66	23.00 ± 15.10	23.67 ± 15.57	30.33 ± 17.16	0.110
**WBC (10^9^/L)**	9.21 ± 2.30	9.62 ± 3.72	9.77 ± 2.17	8.63 ± 2.18	10.87 ± 2.10	9.87 ± 2.97	8.07 ± 1.82	0.546
**Lym (10^9^/L)**	3.94 ± 2.27	3.97 ± 2.07	3.29 ± 1.81	3.60 ± 1.51	4.87 ± 1.75	3.87 ± 2.10	4.17 ± 1.98	0.926
**Lym (%)**	43.87 ± 22.78	47.83 ± 30.31	36.03 ± 22.74	44.73 ± 22.02	45.93 ± 16.35	39.27 ± 19.20	50.10 ± 12.97	0.961
**Mon (10^9^/L)**	0.44 ± 0.32	0.34 ± 0.19	0.29 ± 0.04	0.77 ± 0.23	1.00 ± 0.17	0.97 ± 0.29	0.80 ± 0.10	0.099
**Mon (%)**	4.60 ± 2.33	3.37 ± 0.57	3.03 ± 0.29	8.87 ± 1.91	9.70 ± 2.02	9.87 ± 2.25	9.70 ± 0.52	0.090
**Gran (10^9^/L)**	5.83 ± 2.19	5.31 ± 5.11	6.16 ± 3.53	4.27 ± 3.17	5.00 ± 2.78	4.60 ± 1.61	3.10 ± 0.35	0.049
**Gran (%)**	51.53 ± 20.58	48.80 ± 29.74	60.93 ± 22.98	46.40 ± 23.09	44.37 ± 18.24	48.73 ± 20.94	40.20 ± 12.54	0.301
**RBC (10^12^/L)**	5.85 ± 0.86	5.80 ± 0.71	5.86 ± 0.66	6.41 ± 0.60	6.39 ± 0.39	6.16 ± 0.19	5.80 ± 0.11	1.121
**HGB (g/L)**	127.00 ± 27.73	125.00 ± 28.69	128.00 ± 32.23	128.00 ± 26.63	132.67 ± 19.60	131.00 ± 16.64	118.67 ± 15.70	0.917
**HCT (%)**	40.83 ± 8.69	39.30 ± 8.49	39.07 ± 8.68	44.37 ± 8.16	43.87 ± 6.02	42.33 ± 4.82	40.00 ± 4.47	0.552
**MCV (fL)**	69.33 ± 5.19	67.20 ± 7.13	66.17 ± 8.94	69.07 ± 8.23	68.70 ± 8.55	68.90 ± 8.62	69.07 ± 7.52	0.069
**MCH (pg)**	21.53 ± 1.76	21.33 ± 2.54	21.67 ± 3.69	19.83 ± 2.80	20.73 ± 2.72	21.30 ± 3.03	20,37 ± 2.66	0.067
**PLT (10^9^/L)**	422.00 ± 299.52	415.33 ± 298.26	482.00 ± 319.24	556.33 ± 340.35	536.33 ± 334.32	483.67 ± 296.48	482.00 ± 336.72	0.017
**MPV (fL)**	10.70 ± 0.98	10.53 ± 1.07	10.43 ± 1.32	7.87 ± 0.78	7.90 ± 1.06	7.73 ± 1.34	7.20 ± 1.14	0.018

**(B):** 

Markers	Days after first injection of normal saline							
	0	3	10	20	40	60	75	*p* value
**ALT (U/L)**	79.33 ± 42.67	90.00 ± 51.10	68.00 ± 31.10	49.00 ± 19.92	23.33 ± 10.41	31.00 ± 21.66	39.33 ± 17.67	0.035
**AST (U/L)**	44.00 ± 25.16	80.67 ± 37.86	28.67 ± 10.02	32.00 ± 2.65	25.33 ± 6.66	26.33 ± 5.86	27.67 ± 3.79	0.451
**WBC (10^9^/L)**	7.18 ± 1.62	6.41 ± 2.20	6.24 ± 1.55	6.40 ± 2.39	7.10 ± 1.47	9.17 ± 0.64	6.87 ± 1.10	0.754
**Lym (10^9^/L)**	2.39 ± 1.33	3.23 ± 1.57	2.36 ± 1.16	2.57 ± 0.95	2.60 ± 0.75	2.80 ± 0.87	2.83 ± 1.12	0.038
**Lym (%)**	31.70 ± 14.81	43.40 ± 9.55	36.47 ± 10.90	40.00 ± 4.39	36.30 ± 6.22	32.13 ± 12.63	44.47 ± 5.33	0.014
**Mon (10^9^/L)**	0.45 ± 0.22	0.42 ± 0.29	0.47 ± 0.15	0.53 ± 0.21	0.73 ± 0.21	0.73 ± 0.12	0.60 ± 0.10	0.059
**Mon (%)**	6.13 ± 2.14	6.00 ± 0.96	7.60 ± 2.04	8.70 ± 2.70	10.63 ± 1.05	8.50 ± 1.55	9.90 ± 1.15	0.015
**Gran (10^9^/L)**	4.35 ± 0.98	2.70 ± 0.54	3.41 ± 0.58	3.30 ± 1.39	3.77 ± 0.86	5.53 ± 1.53	2.80 ± 0.40	0.129
**Gran (%)**	62.17 ± 17.15	44.63 ± 11.46	55.93 ± 12.18	51.30 ± 12.18	53.07 ± 6.98	59.37 ± 14.01	45.63 ± 4.37	0.007
**RBC (10^12^/L)**	7.05 ± 1.58	6.85 ± 0.848	6.48 ± 1.33	6.37 ± 1.00	6.59 ± 1.28	6.90 ± 0.27	6.80 ± 0.56	0.007
**HGB (g/L)**	155.67 ± 21.13	149.67 ± 14.67	144.00 ± 18.19	136.67 ± 15.18	146.67 ± 20.50	159.00 ± 2.65	142.67 ± 19.73	0.030
**HCT (%)**	50.50 ± 7.73	46.70 ± 5.99	45.47 ± 6.69	46.20 ± 5.12	47.77 ± 7.56	50.33 ± 0.29	46.93 ± 6.90	0.005
**MCV (fL)**	72.30 ± 4.90	70.30 ± 3.32	70.67 ± 3.78	72.90 ± 3.08	72.97 ± 3.16	73.97 ± 2.58	72.63 ± 2.39	0.699
**MCH (pg)**	22.33 ± 1.87	22.63 ± 1.84	22.43 ± 1.65	21.50 ± 1.20	22.37 ± 1.19	23.03 ± 1.00	22.03 ± 0.81	0.994
**PLT (10^9^/L)**	374.33 ± 72.67	350.67 ± 39.02	346.00 ± 37.16	397.00 ± 74.48	413.67 ± 81.59	420.33 ± 9.71	327.33 ± 30.17	0.929
**MPV (fL)**	10.40 ± 0.53	10.40 ± 0.10	10.40 ± 0.17	8.13 ± 0.32	8.50 ± 0.17	7.90 ± 0.17	8.30 ± 0.26	0.026

Abbreviations: ALT, alanine transaminase; AST, aspartate transaminase; WBC, white blood cells; Lym, lymphocytes; Mon, monocytes; Gran, granulocytes; RBC, red blood cells; HGB, haemoglobin; HCT, haematocrit; MCV, mean corpuscular volume; MCH, mean corpuscular haemoglobin; PLT, platelets; MPV, mean platelet volume.

All animals had complete blood count and liver function monitored during treatment to assess side-effects of the protein (Table 2). Weights of macaques were relatively constant (Table 3). Several hematological parameters were found lower than clinical reference [19] which might be related to SIV infection (Table 4) but no major immune dysregulation or toxicity was detected.

**Table 3:** Weights of rhesus macaques administered with His-TAT-MOD during treatment period. Data are presented as mean ± SD (*n* = 3).

Days after first injection	Weight (kg)
0	6.43 ± 1.30
17	6.18 ± 1.50
58	6.21 ± 1.15
70	6.61 ± 1.83

**Table 4:** T cell parameters of rhesus macaques in treatment and control groups compared with clinical references. Reference values were obtained from rhesus macaques in good health condition [19]. N.D. = not determined.

Markers	His-TAT-MOD	Control	Reference values
Red blood cell (10^12^/L)	4.86–7.10	5.41–8.81	7.0 ± 0.6
White blood cell (10^9^/L)	6.69–13.83	4.10–9.90	14.58 ± 2.35
Lymphocyte (10^9^/L)	1.80–6.80	0.90–4.61	9.27 ± 2.1
CD4+ T cell (10^9^/L)	0.46–1.55	0.25–1.22	N.D.
CD8+ T cell (10^9^/L)	0.25–2.37	0.25–2.37	N.D.

## 3. Discussion

Apart from the potent cytotoxicity caused by rRNA depurination, RIPs are also well-known for inhibition of viruses, with anti-HIV activity attracting the most attention. TCS was reported to suppress HIV replication by lowering the HIV-1 RNA level and production of viral protein p24 in HIV-infected cells [20], while MAP30 and GAP31 inhibited catalytic activity of HIV-1 integrase as well as p24 antigen expression [21]. Our group previously reported that the active atypical type 3 maize RIP inhibited viral replication in HIV-1 acutely infected cell lines [18]. In this report, we proceeded to test if maize RIP also possesses anti-HIV activity *in vivo*.

We tested the antiviral activity of maize RIP *ex vivo* on SHIV89.6- infected macaque PBMC and found an interesting phenomenon in PBMC treated with the active recombinant maize RIP, His-TAT-MOD. Normally, PBMC isolated from SHIV89.6-infected macaques die after culture for 7–10 days and have the cell viability decreased by 50% compared to uninfected PBMC probably because of lysis effect caused by viral replication. However, treatment of His-TAT-MOD improved the cell survival of infected cells by 50% and 100% at the non-cytotoxic doses of 6.75 and 3.38 µM, respectively whereas similar effect was not observed in uninfected macaque PBMC (Figure 1). The enhanced cell survival upon maize RIP treatment may be related to the viral suppression of the protein which in turn alleviates cell death resulted from the lytic process. The low cell viability of both infected and uninfected PBMC at dosage of 13.50 µM is possibly due to the cytotoxicity of His-TAT-MOD. We further tested the antiviral activity of maize RIP on SIV infected macaque PBMC *in vitro* and the results showed that His-TAT-MOD suppressed the viral p27 antigen production with EC_50_ at 5.53 µM on SHIV89.6- and 11.23 µM on SIVmac239-infected cells, respectively (Table 1). This finding was consistent with aforementioned results that His-TAT-MOD conferred protection against SIV-induced lysis and improved the cell survival of SHIV89.6-infected macaque PBMC at a similar concentration, suggesting the maize RIP is effective in suppressing SIV/SHIV replication. Based on these positive observations, we proceeded to examine the *in vivo* antiviral efficacy of maize RIP using non-human primate model.

SIV-infected rhesus macaque is a popular model because rhesus macaque exhibits similar physiological and immune responses as human [22] and HIV has been postulated to originate from SIV through cross-species transmission [23,24,25]. Upon SIV infection, rhesus macaques develop acquired immunodeficiency syndrome (AIDS)-like conditions and finally die of lymphomas or opportunistic infections as HIV-infected humans [26,27]. Our group has reported that compared with Indian rhesus macaques, Chinese rhesus macaques might be a better model used for AIDS research [28]. SHIV89.6-infected Chinese rhesus macaques are therefore used to further evaluate the antiviral efficacy of maize RIP *in vivo*. SHIV89.6 is an engineered hybrid virus capable of replicating to high level during primary infection in rhesus monkeys. This chimeric virus is composed of a viral core derived from SIVmac239 and auxiliary (Tat, Rev and Vpu) and envelope proteins derived from HIV-189.6 [29]. As HIV is originated from SIV, the inhibition on SHIV replication of maize RIP proteins assessed by plasma SIVmac p27 levels here is indicative of their anti-HIV activity.

SHIV-infected macaques were administered with His-TAT-MOD for eight consecutive weeks and SHIV replication was monitored by plasma viral load. The treated macaques showed a decreasing viral RNA level during treatment period with a maximal of one-third reduction (*p* < 0.01 compared to control group) and the viral load returned to a level comparable to the pre-treatment value upon withdrawal of the protein. Though the reducing effect of maize RIP was transient, it did confirm the antiviral activity of the maize RIP *in vivo*. Plasma viral load has been shown to be predictive of AIDS progression and death [30,31,32]. It was reported that a 75% decrease in HIV-1 RNA by zidovudine treatment contributed significantly to delaying AIDS development [33] while a 10-fold decrease in viral load lowered the risk of death by 50% [34]. By reducing the level of viral RNA in infected individuals, it is possible to slow down disease development. Thus, the negative correlation between His-TAT-MOD treatment and viral load suggests the potential of maize RIP as an anti-HIV agent. There was, however, no significant increase in the CD4+ cells (data not shown). This may be due to the short duration of treatment and mild effect of the protein, which was insufficient for the immune system to recover. With several anti-HIV RIP, namely TCS, reported to elicit immune responses in clinical tests [13], we examined the drug safety of maize RIP by monitoring various hematological parameters and liver function during protein administration. No sign of inflammation or liver toxicity was observed throughout the *in vivo* test, showing that maize RIP is safe.

HIV infection is commonly treated by highly active antiretroviral therapy (HAART) which involves the combination of three or more drugs inhibiting at least two viral replication steps. However, long-term use of the inhibitors leads to emergency of resistant HIV strains showing mutations in protease [35] and reverse transcriptase [36]. The resistant strains lower the efficacy of HAART and are found involved in about one-tenth of new infections in US and Europe [37]. Therefore, it is essential to explore new ways to combat the virus. Our work shows that the active form of maize RIP is promising to be further developed into an anti-HIV drug.

## 4. Materials and Methods

### 4.1. Cloning, Expression and Purification of Maize RIP Variants

**Figure 3:**
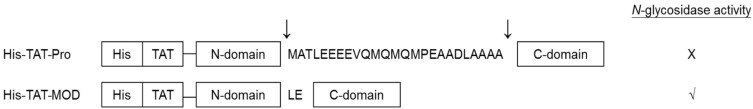
Schematic diagram of maize RIP variants. The artificial maize RIP precursor (His-TAT-Pro) contains a 25aa internal inactivation segment with the native *N*- and *C*-terminal regions removed. The inactivation region is naturally cleaved by maize protease in maize at sites denoted by “↓”. His-TAT-MOD is the active form with the *N*- and *C*-terminal domains fused by a ‘LE’ linker.

TAT-Pro and TAT-MOD were constructed as described previously (Figure 3) [18]. *N*-terminal His-tagged variants His-TAT-Pro and His-TAT-MOD were generated by polymerase chain reaction (PCR) using overlapping primers and Phusion DNA polymerases (Finnzymes). DNA products were cloned into expression vector pET3a and then sequenced to ensure correct mutagenesis. Proteins were expressed in LB using *Escherichia coli* strain C41 (DE3) at 25 °C. Protein expression was overnight induced by 0.4 mM IPTG. Cell pellet was sonicated in 20 mM phosphate buffer, 1.5 M NaCl, 50 mM imidazole, pH 7.8 and supernatant was loaded to a 5 mL HisTrap High Performance column (GE Healthcare) for affinity purification. Protein was eluted by 20 mM phosphate buffer, 300 mM NaCl, 300 mM imidazole, pH 7.8. The elute was then loaded to Superdex 75 (GE Healthcare) pre-equilibrated with 20 mM phosphate buffer, 200 mM NaCl, 5% glycerol, pH 7.4 for size-exclusion chromatography. The purified protein was concentrated and stored at −80 °C.

### 4.2. Animals for Experiment

Ten male rhesus macaques (*Macaca mulatta*) used in this study were colony-bred rhesus macaques of Chinese origin and carried out according to the regulations of the American Association for Assessment and Accreditation of Laboratory Animal Care (AAALAC) at the Kunming Primate Research Center, Kunming Institute of Zoology, Chinese Academy of Sciences (CAS). All experimental procedures were performed at the ABSL-3 laboratory according to the guidelines approved by the Ethics Committee of Kunming Institute of Zoology (Approval Number: SYDW-2012018). All animals were screened and confirmed to be negative for SIV, simian type D retrovirus (SRV), simian T lymphotropic virus (STLV) type I and II by antibody ELISA and PCR. Four macaques (6–8 years old) were used for PBMC isolation and six (6–8 years old) for *in vivo* antiviral study. Macaques for *in vivo* study were infected with 1000 TCID_50_ SHIV89.6.

### 4.3. Cells and Viruses

PBMC were isolated from SHIV89.6-infected and healthy macaques by Ficoll-hypaque centrifugation (GE Healthcare Life Sciences, Pittsburgh, PA, USA) and maintained in RPMI-1640 medium supplemented with 10% heat-inactivated newborn calf serum (Gibco, Shanghai, China). Prior to use, cells were activated by incubating PBMC in complete medium with 10 µg/mL concanavalin A (Con A) and 50 U/mL recombinant IL-2. Cells were kept at 37 °C for 72 h in a humidified incubator with 5% CO_2_ for activation.

### 4.4. Cytotoxicity Assay

Cell viability was assessed by trypan blue stain counting. Macaque PBMC were seeded on 96-well plate at density of 5 × 10^5^ cells/well and treated by different concentration of proteins. Cells incubated without protein were included as negative control. After incubated at 37 °C for 7 d, 0.4% trypan blue in PBS was added and the number of live cells was counted under microscope. Proteins 50% cytotoxicity concentration (CC_50_) was determined.

### 4.5. Viral Antigen Reduction Assay

Macaque PBMC were seeded on 96-well plate at density of 5 × 10^5^ cells/well and infected with SIVmac239 and SHIV89.6 at MOI of 0.02 and 0.10, respectively. Cells were then treated by different concentration of proteins. After incubated at 37 °C for 7 d, culture supernatants were collected for viral antigen determination using SIV p27 Antigen ELISA (ZeptoMetrix, New York, NY, USA). Protein EC_50_ was determined.

### 4.6. Protection of Infected Macaque PBMC from Lysis ex vivo

PBMC were isolated from SHIV89.6-infected (#04331, #06003 and #06311) or healthy macaques (#06089) and seeded on 96-well plate at density of 5 × 10^5^ cells/well. After treated by different concentration of proteins for 7 d, cell viability was assessed by trypan blue exclusion counting. Cells incubated without protein were included as negative control. The percentage of protection was calculated by comparing cell number of treated sample with that of control and paired T-test was used for statistical analysis.

### 4.7. Administration of Maize RIP and Sample Collection

SHIV89.6-infected macaques (weighed 5–14 kg) were randomly assigned to either treatment (#06003, #06025 and #06311) or control groups (#05845, #04097 and #04331). Macaques were administered weekly with either His-TAT-MOD at dose of 50 µg/kg of body weight or normal saline as negative control by intravenous injection at hind legs for eight consecutive weeks. Blood was collected at designated time-points (before treatment and 3, 10, 20, 40, 60 and 75 day after the first injection) for plasma viral load determination. Basic clinical examinations on weight, complete blood count (CBC) testing and liver function were also done throughout treatment period. CBC testing was performed using the Auto Hematology Analyzer (Mindraya, Shenzhen, Guangdong, China) and liver function was detected in First People’s Hospital of Yunnan Province according to the manufacturer’s protocol (SYDW-2012018).

### 4.8. Plasma Viral Load Determination

Plasma viral load was determined by TaqMan real-time RT-PCR(Takara, Dalian, Liaoning, China). Briefly, plasma was separated from whole blood collected in EDTA-K2-containing tubes. Viral RNA was then extracted using the High Pure Viral RNA Kit (Roche Life Science, Indianapolis, IN, USA) according to the manufacturer’s instructions and stored at −80 °C until use. A two-step RT-qPCR assay was done using the PrimeScript™ (Takara, Dalian, Liaoning, China) RT reagent Kit and Premix ExTaq™ (Takara, Dalian, Liaoning, China) on 7500 Fast Real-Time PCR System (Applied Biosystems, Foster City, CA, USA). The detection limit was 200 copies/mL. The 20 µL-PCR reaction contained 10 μL of Premix Ex Taq(Takara, Dalian, Liaoning, China), 0.4 μL of ROX reference Dye II, 200 nM of primer pair, 100 nM of TaqMan probe and 2 μL of standard or samples. Probe and primers were designed to bind within the conserved SIVmac gag region. The primers were 5'-TCGGTCTTAGCTCCATTAGTGCC-3' and 5'-GCTTCCTCAGTGTGTTTCACTTTC-3' and the TaqMan probe was 5'-CTTCTGCGTGAATGCACCAGATGACGC-3'. The probe had 6-carboxyfluorescein (FAM) at 5' end as fluorescence reporter dye and 6-carboxytetramethylrhodamine (TAMRA) at 3' end as quencher dye. The control template was an *in vitro* transcript of pGEM-4ZSIVgag357 carrying the SIV gag fragment from SIVmac239 and prepared from plasmid p239SpSp5' provided by Bin Gao of University College London. A serial dilution of control template was included to construct standard curve.

### 4.9. Flow Cytometry

Whole blood was collected from macaques in EDTA tubes and the number of T-cell subsets were analyzed by flow cytometry as previously described [28]. Undiluted blood (50 μL) was stained in TruCount tubes (BD Biosciences, San Jose, CA, USA) with mouse anti-human monoclonal antibodies selected for efficient cross-reaction with Chinese rhesus macaques. The T lymphocytes and subsets were labeled with FITC-CD8 (Miltenyi Biotec, Teterow, Germany, Clone BW135/80), PE-CD3 (Miltenyi Biotec, Teterow, Germany, Clone 10D12) and PerCP-CD4 (BD Biosciences, San Jose, CA, USA, Clone L200). Cells were then loaded to a three-color flow cytometry, FACScalibur (Becton Dickinson, San Jose, CA, USA) and analyzed by Cell Quest software (Tree Star Software, Palo Alto, CA, USA).

### 4.10. Statistical Analysis

Data were analyzed using the Statistical Package for the Social Sciences (SPSS) software version 17.0 (SPSS Inc., Chicago, IL, USA). Virus load was evaluated by ANOVA. CBC testing, liver function and lymphocyte count were analyzed by independent samples *T*-test. *p* < 0.05 represents statistically difference. Trends in time were analyzed by multivariate linear regression.
